# Glucagon-like Peptide-1 Receptor Agonists and Diabetic Kidney Disease: From Bench to Bed-Side

**DOI:** 10.3390/jcm13247732

**Published:** 2024-12-18

**Authors:** Aly M. Abdelrahman, Alaa S. Awad, Irtiza Hasan, Emaad M. Abdel-Rahman

**Affiliations:** 1 Department of Pharmacology & Clinical Pharmacy, College of Medicine and Health Sciences, Sultan Qaboos University, Alkhod 123, Oman; abdelrahman@squ.edu.om; 2 Division of Nephrology, University of Florida, Jacksonville, FL 32209, USA; alaa.awad@jax.ufl.edu (A.S.A.); md.irtiza.hasan@jax.ufl.edu (I.H.); 3 Division of Nephrology, University of Virginia, Charlottesville, VA 22908, USA

**Keywords:** GLP-1, DKD, albuminuria, mechanism of action, animal studies, human studies, safety

## Abstract

Glucagon-like peptide-1 (GLP-1) receptor agonists are currently available for the management of type 2 diabetes mellitus. They have been shown to help with diabetic kidney diseases through multiple mechanisms. In this review, we will shed light on the different mechanisms of action through which GLP-1 receptor agonists may achieve their roles in renal protection in diabetics, both in animal and human studies, as well as review the renal outcomes when using these drugs and their safety profile in diabetic patients.

## 1. Introduction

Diabetic kidney disease (DKD) is a serious microvascular complication affecting approximately 20–50% of individuals with diabetes mellitus (DM) and is the leading cause of End Stage Kidney Disease (ESKD) worldwide [1]. DM is increasing worldwide in both developed and developing countries. While in Africa, Europe, South East Asia, and South and Central America the proportion of diabetics is 9%, that proportion increases to 13%, 14%, and 17% in the West Pacific, North America and the Caribbean, and Middle East and North Africa, respectively. It is further predicted that this proportion of diabetics will continue to increase at a much greater rate in less than 25 years [1].

It is estimated that, in diabetic patients, the prevalence of chronic kidney disease (CKD) ranges from 27% to 84% depending on the location, with the annual incidence of albuminuria measuring 2–8% and estimated glomerular filtration rate (eGFR) < 60 mL/min/1.73 m^2^ amounting to 2–4% [1].

In recent decades, several medications have been developed to delay or reverse the progression of DKD, such as renin aldosterone angiotensin system (RAAS) blockers, sodium–glucose cotransporter 2 inhibitors (SGLT2is), and the nonsteroidal selective mineralocorticoid receptor antagonist finerenone. Another group of drugs were evaluated to assess their role in managing patients with DKD: glucagon-like peptide-1 receptor agonists (GLP-1RAs).

Glucagon-like peptide 1 (GLP-1) belongs to the incretin group of gastrointestinal (GI) hormones that was demonstrated to help patients with diabetes mellitus type II (DM II) as well as obesity [2]. Recent studies have also shown that GLP-1 might be helpful in patients with DKD, where it exerts its effects via GLP-1 receptors (GLP-1R). Several drugs have been studied that act on the GLP-1R and are termed GLP-1RAs.

In this review, we aim to explore the mechanism of action of GLP-1RAs, highlighting the preclinical and clinical evidence regarding their potential role in diabetic kidney disease (DKD). We will also address their safety profile and the common side effects of these drugs.

## 2. Glucagon-like Peptide 1

Glucagon-like peptide 1 (GLP-1) is an incretin hormone secreted by intestinal L cells and pancreatic α-cells [3]. GLP-1 is secreted and released by enteroendocrine L cells within the terminal ileum, colon, and brainstem neurons [4,5,6,7] through the action of prohormone convertase 1 [8] in response to meals. It has a rapid half-life and is degraded by dipeptidyl peptidase 4 (DPP-4) [4,5].

GLP-1 exerts various effects on pancreatic beta cells, such as enhancement of glucose-dependent insulin secretion, acceleration of beta cell proliferation, and inhibition of beta cell apoptosis, which occur through activation of GLP-1 receptors (GLP-1Rs) in the pancreas [9]. GLP-1 also inhibits glucagon secretion and decreases appetite and food intake [10]. In the GI tract and hypothalamus, GLP-1 inhibits motility, gastric emptying, and central regulation of feeding, resulting in body weight loss [11]. Thus, GLP-1, through these actions, helps to improve glucose metabolism [12].

In addition to GLP-1R expression in the pancreas and the GI tract, GLP-1Rs are also expressed in other tissues, such as the heart, brain, and kidneys [9]. In the rat and human kidneys, GLP-1R was found in the preglomerular vasculature; however, some studies suggested that GLP-1R was also expressed in the proximal tubules [9]. Hviid and Sørensen [13] reviewed the results of the studies that examined renal GLP-1R distribution and showed that there were conflicting results. They suggested that the conflict in the GLP-1R distribution might be due to the used method or tissue. They concluded that, by using specific antibodies, the presence of GLP-1R in the renal vasculature was confirmed but not in the renal tubules.

## 3. Glucagon-like Peptide Receptor Agonists

GLP-1 R agonists (GLP-1RAs) have been approved for the treatment of patients with type 2 DM [14], acting by activating GLP-1Rs [15]. They have been recommended for patients with DM who have not met their glycemic targets despite therapeutic optimization. GLP-1 RAs have been shown to be superior to some other hypoglycemic drugs. Rodbard et al. compared the efficacy of either oral semaglutide (*n* = 400) or the sodium–glucose cotransporter 2 inhibitor empagliflozin (*n* = 387) in lowering hemoglobin A1C (HbA1c) in diabetics uncontrolled on metformin. They demonstrated the superior efficacy of oral semaglutide to empagliflozin [16]. GLP-1 RAs have also been shown to have other beneficial effects. A post hoc analysis of the cardiovascular outcome trials (CVOT) [17] indicated possible benefits of GLP-1RAs in delaying DKD progression [18].

GLP-1RAs are classified as exendin-4- or human-GLP-1-based compounds [19]. They are divided into short-acting (exenatide and lixisenatide) or long-acting (dulaglutide, liraglutide, exenatide long-acting release, and semaglutide) [20]. Human-GLP-1-derived dulaglutide, liraglutide, and semaglutide are not excreted via the kidneys and can be used down to an eGFR of 15 mL/min/1.73 m^2^. Exenatide and lixisenatide are eliminated by the kidneys and are contraindicated below an eGFR of 30 mL/min/1.73 m^2^ due to the risk of accumulation and toxicity [18]. Both experimental and clinical studies have demonstrated the renoprotective actions of GLP-RAs.

## 4. Mechanism of Renoprotective Action from Preclinical and Clinical Studies (Table 1)

It is not clear how GLP-1RAs evoke their specific renoprotective effects. However, their renoprotective effects might be due to reductions in blood pressure, body weight, and plasma glucose (Table 1). In addition, GLP-1RAs also reduce inflammation, reactive oxygen species, and endothelial dysfunction, leading to a reduction in the development of albuminuria [9]. Mosenzon et al. analyzed results of high-sensitivity C-reactive protein (hsCRP) as an inflammatory marker from SUSTAIN 3 and PIONEER 1, 2 and 5 studies that used semaglutide subcutaneously and orally, respectively, in diabetic subjects (*n* = 2482). The results of hsCRP using semaglutide were compared (*n* = 1328). The investigators showed that semaglutide significantly reduced hsCRP more than its counterparts (*p* < 0.01), except versus placebo, pointing to anti-inflammatory effects of GLP-1RAs [21].

**Table 1:** Possible mechanisms of renoprotective action of glucagon-like peptide receptor agonists.

Reduction in oxidative stress [9,19]
Reduction in inflammation [9,19]
Natriuresis/Diuresis [9,19]
Reduction in intraglomerular pressure [19]
Reduction in hyperglycemia [9,19]
Reduction in blood pressure [9,19]
Reduction in obesity [9,19]
Reduction in endothelial dysfunction [9]

**Figure 1:**
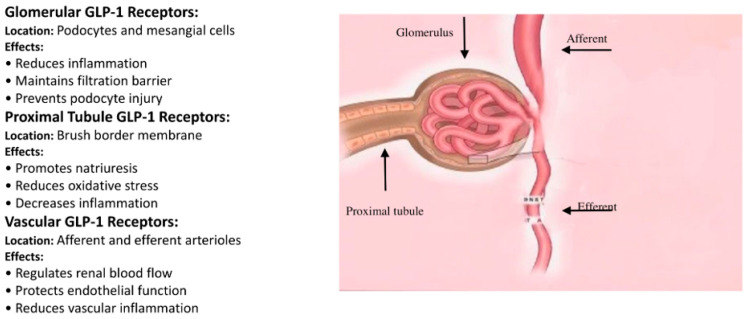
Location and Effects of GLP-1 in the Nephron.

Further actions of GLP-1RAs that may aid renal protection include reducing hyperlipidemia, induction of natriuresis, and diuresis [18]. Preclinical studies have shown that exendin 4 (10 µg/kg/day) for 8 weeks reduced glomerular hyperfiltration in streptozotocin-induced diabetes in Sprague Dawley rats [11]. On the other hand, the GLP-1RA effects on renal hemodynamics remain controversial. While GLP-1RAs were shown to have direct vasodilatory action on eGFR and increasing filtration, they can also have an opposing effect by inhibiting factors causing hyperfiltration and by impairing nitric oxide (NO)-dependent vasodilatory actions. This may lead to a neutral effect or decrease eGFR with GLP-1RAs [18]. Other studies failed to demonstrate these changes in GFR with GLP-1RAs. A double-blind, randomized, placebo-controlled trial by Tonneijck and colleagues assessed the GFR and effective renal plasma flow (ERPF) along with other renal hemodynamic parameters, such as filtration fraction, glomerular hydrostatic pressure, and vascular resistance, in the afferent and efferent renal arterioles following the administration of either exenatide (*n* = 24) or placebo (*n* = 28) intravenously in overweight type 2 diabetic patients [22]. The researchers demonstrated that exenatide alone does not have an acute effect on renal hemodynamics in the population studied [22]. Similar results were noted when comparing lixisenatide’s effect on postprandial glomerular hemodynamics versus insulin-glulisine (iGlu) in diabetic patients, showing no effect of lixisenatide on renal hemodynamics compared to iGlu in an eight-week study period [23]. In summary, while preclinical and clinical data have shown a role for GLP-1RAs in renal protection, data remain controversial as to the exact mechanisms of renoprotection and the sites of action in the nephron (Table 1, Figure 1).

## 5. Renal Outcome Effects of GLP-1 RAs

### 5.1. Preclinical Studies (Table 2)

#### 5.1.1. Liraglutide

A liraglutide (0.2–0.6 mg/kg/day) treatment for 8 weeks reduced the renal pathologic findings and urinary albumin in early-phase DKD in spontaneously diabetic Torii fatty rats by preventing glomerular endothelial abnormality and preservation of autophagy. These renoprotective effects noted were independent of blood glucose and blood pressure levels [24]. Liraglutide (100 µg/kg and 200 µg/kg/day) treatment for 8 weeks delayed the progress of diabetic nephropathy in Sprague Dawley rats that were fed a high-sugar and high-fat diet and received streptozotocin by reducing endoplasmic reticulum stress [25]. The effect of liraglutide on delaying the progression of diabetic nephropathy was confirmed in another study through other mechanisms. Liraglutide (0.3 mg/kg twice daily) was administered for 8 weeks in streptozotocin-induced diabetes in Sprague Dawley rats, which prevented the progression of diabetic nephropathy by modulating the crosstalk between transient receptor potential canonical 6 (TRPC6) and nicotinamide adenine dinucleotide phosphate (NADPH) oxidases [26]. In high-fat–high-sugar-fed Sprague Dawley rats that were treated with streptozotocin, liraglutide (0.6 mg/kg/day) for 12 weeks reduced albuminuria, and the authors suggested that liraglutide may have a renoprotective effect in DKD through its effect on the Micro RNA-34A (miR-34a)/sirtuin 1 (SIRT1) pathway [27]. In Zucker diabetic fatty (ZDF) rats, liraglutide (200 µg/kg/12 h) for 9 weeks decreased urinary albumin and attenuated renal pathological changes. This renoprotective effect was due to the activation of autophagy by regulating the adenosine monokinase (AMP)-activated protein kinase-mammalian target of the rapamycin pathway [28]. Huang and colleagues [29] showed that administration of liraglutide (200 µg/kg/12 h) for 8 weeks to streptozotocin-induced diabetic Sprague Dawley rats improved renal function without lowering blood glucose levels and ameliorated glomerular histopathological changes. They further showed that liraglutide reduced the production of glomerular extracellular matrix proteins by enhancing Wnt/β-catenin signaling [29]. In Sprague Dawley rats fed a high-sugar and high-fat diet and injected with low-dose streptozotocin to induce type 2 diabetes, liraglutide (0.2 mg/kg/12 h) for eight weeks had a renoprotective effect by activating forkhead box protein O1 (FoxO1) [30]. Meanwhile, in Wistar rats with streptozotocin-induced diabetes mellitus, liraglutide (0.3 mg/kg/12 h) for 12 weeks had a direct beneficial effect on diabetic nephropathy by improving endothelial nitrous oxide synthase (eNOS) activity via downregulating the nuclear factor (NF)-κB (NF-κB) inflammatory pathway [31]. In streptozotocin-induced diabetic Wistar rats, liraglutide (0.3 mg/kg/12 h) for four weeks reduced oxidative stress, expression of NAD(P)H oxidase components, transforming growth factor-beta (TGF-β), fibronectin in renal tissues, and urinary albumin excretion [32].

**Table 2:** Effects of glucagon-like peptide-1 receptor agonists on experimental animal models of diabetic nephropathy.

Drug	Animal	Effects of GLP-1 Receptor Agonists	Ref.
**Liraglutide**	- *Spontaneously diabetic Torii fatty rats.*	- *Reduced renal pathologic findings and urinary albumin in in early-phase diabetic kidney disease by preventing glomerular endothelial abnormality and preservation of autophagy*	[24]
	- *Sprague Dawley rats fed a high-sugar and high-fat diet and received streptozotocin.*	- *Delayed the progress of diabetic nephropathy by reducing endoplasmic reticulum stress.*	[25]
	- *Streptozotocin-induced diabetes in Sprague Dawley rats.*	- *Prevented the progression of diabetic nephropathy by modulating the crosstalk between TRPC6 and NADPH oxidases.*	[26]
	- *High-fat–high-sugar-fed Sprague Dawley rats that were treated with streptozotocin.*	- *Reduced albuminuria, renoprotective effect in DN through its effect on miR-34a/SIRT1 pathway.*	[27]
	- *Zucker diabetic fatty (ZDF) rats*	- *Decreased urinary albumin and attenuated renal pathological changes. Renoprotective effect due to activation of autophagy by regulating AMP-activated protein kinase-mammalian target of rapamycin pathway.*	[28]
	- *Streptozotocin-induced diabetic in Sprague Dawley rats*	- *Improved renal function and ameliorated glomerular histopathological changes. Liraglutide reduced the production of glomerular extracellular matrix proteins by enhancing Wnt/β-catenin signaling.*	[29]
	- *Sprague Dawley rats fed with high sugar and high-fat diet and injected with low-dose streptozotocin*	- *Had a renoprotective effect by the activation of forkhead box protein O1 (FoxO1).*	[30]
	- *Wistar rats with streptozotocin-induced diabetes mellitus.*	- *Had a direct beneficial effect on diabetic nephropathy by improving eNOS activity via downregulating NF-κB.*	[31]
	- *Streptozotocin-induced diabetic Wister rats.*	- *Reduced oxidative stress, expression of NAD(P)H oxidase components, TGF-β, and fibronectin in renal tissues and urinary albumin excretion.*	[32]
	- *Diabetic Ins2Akita mice (C57BL/6-Ins2Akita/J).*	- *Reduced albuminuria, glomerulosclerosis, and glomerular basement membranous thickness kidney-protective effect due to dampening the receptor for advanced-glycation-end-product-induced inflammation.*	[3]
	- *C57BL/6J mice fed high-fat diet and treated with streptozotocin.*	- *Reduced urinary protein, attenuated podocyte damage, and glomerular injury via reducing NLRP3-mediated inflammation.*	[33]
	- *C57BLKS/J db/db diabetic mice.*	- *Induced* *browning of white adipose tissue, which protects podocytes by decreasing TNF-α secretion and activation of PI3K)/AKT pathway.*	[34]
	- *Diabetic-nephropathy-prone KK/Ta-Akita mice.*	- *Reduced albuminuria and mesangial expansion.*	[35]
**Exantide**	- *Wistar rats fed high-fat diet and followed by injection of streptozotocin.*	- *Improved renal function through correction of glycolipid intolerance as well as reducing oxidative stress*.	[36]
	- *Sprague Dawley rats injected with streptozotocin.*	- *Ameliorated renal injury through decreased oxidative stress and inflammatory response in renal tissue.*	[37]
	- *Streptozotoci-induced diabetes in Sprague Dawley rats.*	- *Reduced albuminuria, glomerular hyperfiltration, glomerular hypertrophy, and mesangial matrix expansion.*	[11]
	- *C57BL/6J mice fed high-fat diet and were injected with streptozotocin.*	- *Reduced urinary albumin and attenuated the progress of diabetic nephropathy via activation of renal AMP-activated protein kinase.*	[38]
	- *Diabetic apoE^−/−^ mice (ApoE^−/−^ DM) fed high-fat diet and injected with streptozotocin.*	- *Increased ABCA1 expression in glomerular endothelial cells and attenuated renal lipid accumulation, inflammation, and proteinuria.*	[39]
	- *Streptozotocin-induced diabetes in BALB/c mice.*	- *Decreased renal tubular injury of diabetic nephropathy by decreasing oxidative stress and inflammation.*	[40]
**Semaglutide**	- *db/db UNx-ReninAAV mice.*	- *Reduced albuminuria and glomerulosclerosis severity and hypertension.*	[41]
	- *Lepr db/db (db/db) mice, a diabetic nephropathy model.*	- *Decreased collagen deposition, attenuated kidney fibrosis, and kidney injury.*	[42]
**Lixisenatide**	- *Wister rats fed a high-fat diet and injected with streptozotocin.*	- *Has a nephroprotective effect, as shown by improved kidney function and renal histopathology.*	[43]

In diabetic Ins2Akita mice (C57BL/6-Ins2Akita/J), liraglutide (50 mg/kg/day for 20 weeks) reduced albuminuria, glomerulosclerosis, and glomerular basement membranous thickness. The authors suggested that this kidney-protective effect was due to dampening the receptor for advanced-glycation-end-product-induced inflammation and that this is a glucose-independent effect [3]. In C57BL/6J mice fed a high-fat diet and treated with streptozotocin to induce type 2 diabetes, liraglutide (400 µg/kg/day) treatment for 14 weeks reduced urinary protein and attenuated podocyte damage and glomerular injury via reducing nucleotide-binding domain, leucine-rich-containing family, and pyrin-domain-containing-3 (NLRP3)-mediated inflammation [33]. Liraglutide (400 mg/kg/day) treatment for 8 weeks induced browning of white adipose tissue, which protects podocytes by decreasing tumor necrosis factor-alpha (TNF-α) secretion and activation of phosphoinositide 3-kinase (PI3K)/protein kinase B (AKT) pathway in C57BLKS/J db/db diabetic mice [34]. Treatment with liraglutide (200 µg/kg/day) for four weeks ameliorated the progression of nephropathy in a mouse model of progressive diabetic nephropathy (KK/Ta-Akita mice), as shown by the reduced albuminuria and mesangial expansion [35].

#### 5.1.2. Exenatide

In Wistar rats fed a high-fat diet and followed after injection of streptozotocin, exenatide (5 µg/kg/day) for four weeks improved renal function through correction of glycolipid intolerance as well as reducing oxidative stress [36]. In Sprague Dawley rats injected with streptozotocin, exenatide (2, 4, and 8 μg/kg) for 8 weeks ameliorated renal injury through decreased oxidative stress and inflammatory response in renal tissue [37]. Exendin 4 (10 µg/kg/day) for 8 weeks reduced albuminuria, glomerular hyperfiltration, glomerular hypertrophy, and mesangial matrix expansion in streptozotocin-induced diabetes in Sprague Dawley rats [11].

Exendin 4, administered for 8 weeks to C57BL/6J mice fed a high-fat diet and that were injected with streptozotocin, reduced urinary albumin and attenuated the progress of diabetic nephropathy via activation of renal AMP-activated protein kinase [38]. In diabetic apoE^−/−^ mice (ApoE^−/−^ DM) fed high-fat diet and injected with streptozotocin, exendin 4 (1 nmol/kg/day) for 8 weeks increased ABC Transporter A1 (ABCA1) expression in glomerular endothelial cells and attenuated renal lipid accumulation, inflammation, and proteinuria [39]. Sancar-Bas et al. [40] showed that exendin (3 µg/kg) decreased renal tubular injury of diabetic nephropathy by reducing oxidative stress and inflammation in streptozotocin-induced diabetes in BALB/c mice.

#### 5.1.3. Semaglutide

*In db/db* UNx-ReninAAV mice, a model of hypertension accelerated diabetic kidney disease, and semaglutide (30 nmol/kg/day) for 11 weeks reduced hyperglycemia, albuminuria, and glomerulosclerosis severity and hypertension [41]. Semaglutide (120.0 μg/kg/day) injection for eight weeks to Lepr *db*/*db* (*db*/*db*) mice, a diabetic nephropathy model, decreased collagen deposition and attenuated kidney fibrosis and kidney injury [42].

#### 5.1.4. Lixisenatide

In Wistar rats fed a high-fat diet and injected with streptozotocin, lixisenatide (1 nmol/kg/day) for 2 weeks induced a nephroprotective effect, as shown by improved kidney function and renal histopathology [43].

### 5.2. Clinical Studies (Table 3)

Several studies have evaluated the effect of GLP-1RAs on renal outcomes such as albuminuria and progression of renal disease as assessed by the estimated glomerular filtration rate (eGFR) The safety and adverse effects of these drugs were also evaluated.

In a systemic review that included seven trials (*n* = 56,004) using different GLP-1 RA drugs, the researchers demonstrated a reduction in a broad composite of kidney outcomes, progression to ESKD, or death attributable to kidney causes by 17% when receiving GLP-1 RAs [44]. These benefits in renal outcomes noted when using these drugs were mainly due to a reduction in urinary albumin excretion. No increased risk was associated with using these drugs.

Schechter and colleagues studied the effects of GLP-1RAs (exenatide, exenatide extended release, liraglutide, dulaglutide, semaglutide, and lixisenatide) versus basal insulin on albuminuria and composite kidney outcome in diabetic patients (*n* = 3424) with a median follow-up of 81.1 months [45]. In an as-treated analysis, the researchers demonstrated a significant benefit of GLP-1RAs in decreasing the eGFR slope compared to basal insulin [45]. Similarly, Lin and colleagues [46], over a median follow-up of 2.1 years, showed an advantage of using GLP-1RAs (*n* = 759) vs. DPP-4 inhibitors (*n* = 8163).

In pooled data from two major trials, the SUSTAIN 6 trial [47] using semaglutide and the LEADER trial [48] using liraglutide (*n* = 12,637), the researchers showed that both these drugs lowered albuminuria by 24% as compared to the placebo. Both drugs also slowed the decline in the eGFR slope by 0.87 mL/min/1.73 m^2^/year and 0.26 mL/min/1.73 m^2^/year for semaglutide 1.0 mg weekly and liraglutide 1.8 mg weekly, respectively. They further showed that there was a statistically significant decrease in the incidence of 40% (*p* = 0.039) or 50% (*p* = 0.023) eGFR decline, but not for the 30% or 57% decline outcomes. Lastly, the study showed a benefit in composite endpoint, time from randomization to kidney failure, death, and proportional eGFR decline. The renal-protection effect noted when using these drugs was greater in patients with advanced kidney disease (eGFR < 60 mL/min/1.73 m^2^) and those with heavy albuminuria [49].

### 5.3. Individual Drugs

#### 5.3.1. Semaglutide

Tuttle and coworkers [50] conducted a post hoc analysis of data pooled from two studies: SUSTAIN 6 [47] and Pioneer 6 [51]. Both studies were conducted on patients with DM II with high cardiovascular risks. In this post hoc analysis, the researchers focused on the effect of semaglutide on the decline in renal function, comparing semaglutide (*n* = 3239) versus a placebo (*n* = 3241). The researchers demonstrated a significant reduction in the rate of eGFR decline in the semaglutide group in the total population studied in a subgroup with a baseline eGFR 30 to <60 mL/min/1.73 m^2^ and in a subgroup with a baseline eGFR > 60 mL/min/1.73 m^2^ with *p* values < 0.0001, 0.0007 and 0.0083, respectively [50].

A post hoc analysis of the patients (*n* = 8416) that were enrolled in SUSTAIN 1–5, SUSTAIN 7 [52,53,54], and SUSTAIN 6 [47] trials was conducted, aiming to evaluate the effectiveness and safety of once-weekly semaglutide administered in two different doses (0.5 mg and 1.0 mg) compared to the drugs used in these studies (sitagliptin, exenatide, insulin glargine, dulaglutide, and the placebo [55]. While the duration of SUSTAIN studies 1 through 7 ranged between 30 and 104 weeks, the analysis for the post hoc trial was conducted on data pooled up to 30 weeks for eGFR and to week 56 for urine albumin to creatinine ration (UACR) for SUSTAIN 1–5 and SUSTAIN 7, and up to 104 weeks in SUSTAIN 6. The authors showed that both doses of semaglutide demonstrated marked reductions in UACR compared to the placebo [55].

More recently, in 2023, Rossing and colleagues published an article to detail the rationale, design, and baseline data of the FLOW trial [56]. A year later, the results of the trial were published [57,58,59,60]. The FLOW trial, a randomized, double-blind, parallel-group, multinational trial, was conducted on diabetic patients with chronic kidney disease (CKD), aiming to investigate the effects of semaglutide versus a placebo on kidney outcomes. Two groups of patients were included based on the eGFR and proteinuria; the first group had eGFR >50–≤75 mL/min/1.73 m^2^ with UACR > 300 to ≤5000, while the second group had eGFR >25–<50 mL/min/1.73 m^2^ with UACR >100–≤5000. The composite primary endpoint was time to first kidney failure, persistent >50% reduction in eGFR, or death from kidney or cardiovascular (CV) causes [56]. After a median follow-up of 3.4 years, the researchers demonstrated a 24% decrease in the primary endpoint in the semaglutide group (*n* = 1767) compared to the placebo group (*n* = 1766) [57]. The severity of the baseline CKD had no significant effect on the beneficial effects noted in the semaglutide-treated group on the risk of CV death, myocardial infarction, or stroke [58]. To further study the effect of adding SGLT2 inhibitors to semaglutide for patients in the FLOW trial, the researchers found that, regardless of the presence or absence of SGLT2i, semaglutide was beneficial in reducing composite renal outcomes [59].

#### 5.3.2. Liraglutide

Mali and colleagues conducted a meta-analysis using randomized controlled trials that studied the effect of liraglutide on renal function in patients with DN. They reviewed 18 studies (*n* = 1580) and showed that liraglutide efficiently controlled diabetes, overweight, and renal outcomes [61].

A randomized controlled trial by Mann and colleagues [48] (LEADER trial) assessed the renal outcome (new-onset macroalbuminuria, doubling of serum creatinine, ESKD, or renal-related death) in patients (*n* = 9349) with DM II and high cardiovascular risk over a median follow-up period of 3.84 years. The researchers compared liraglutide versus a placebo and showed the renal outcome to be significantly better in the liraglutide group. This result was mainly driven by the fewer new onsets of persistent macroalbuminuria, which occurred in fewer participants in the liraglutide group than in the placebo group.

#### 5.3.3. Dulaglutide

The randomized controlled trial REWIND [62] was a study on patients with DM II (*n* = 9901). In this trial, which had a median follow-up of 5.4 years, the effects of dulaglutide (*n* = 4949) on the renal outcome were compared with those of the placebo (*n* = 4952). The study demonstrated a significant decrease in renal outcomes with dulaglutide compared to the placebo. The study also showed a superior effect of dulaglutide compared to the placebo in decreasing new macroalbuminuria.

Dulaglutide in two doses, 1.5 mg (*n* = 193) and 0.75 mg (*n* = 190), was compared to insulin glargine (*n* = 194) in diabetic patients with CKD stages 3 to 4 (AWARD 7 trial) [63], with the primary outcome being HBA1c and the secondary being eGFR and albuminuria. Dulaglutide in both doses was non-inferior to insulin glargine, with a significant improvement in eGFR in the dulaglutide-treated group. No difference was noted in UACR in both groups.

#### 5.3.4. Efpeglenatide

Another drug studied was efpeglenatide in the AMPLITUDE-O trial [64]. This randomized placebo-controlled trial was conducted in 28 countries over 344 sites (*n* = 4076). The study aimed to assess the effect of efpeglenatide at a dose of 4 or 6 mg versus a placebo in patients with DM II with either a history of cardiovascular disease or current chronic kidney disease with eGFR 25.0–59.9 mL/min/1.73 m^2^ on renal outcome, namely a decrease in kidney function or macroalbuminuria. The follow-up period was 1.81 years. The authors showed that the composite of the renal outcome events was significantly lower in the Efpeglenatide-treated group (*p* < 0.001), with a decrease in the urine albumin ratio by 21% and a mean increase in eGFR by 0.9 mL/min/1.73 m^2^.

In summary, multiple studies have confirmed a renoprotective effect of GLP-1RA drugs in diabetic animals and humans. Most of the renoprotective action of these drugs is attributed to a reduction in UACR.

**Table 3:** Renal outcomes with GLP-1.

Study	Drug	Patients	Renal Outcomes and Results		
			Macroalbuminuria	eGFR	Composite
**SUSTAIN-6 [47,55]** Post Hoc Analysis F/U Median 2.1 years	**Semaglutide** SQ 0.5 and 1.0 mg weekly versus Placebo.	*n* = 3297. DM II, CKD, Cardiovascular or both Semaglutide (*n* = 1648) Placebo (*n* = 1649)	- Reduced incidence with semaglutide 0.97 (0.5 mg) 0.86 1.0 mg 1.32 placebo	- Greater decline with semaglutide until week 16 then less decline weeks 16 to 104	- No difference
**LEADER** [48] Randomized controlled. F/U Median 3.84 years	**Liraglutide** 1.8 mg or maximum tolerated dose versus Placebo.	*n* = 9340, DM II, CKD 4, Cardiovascular risk Liraglutide (*n* = 4668) Placebo (*n* = 4672)	- Significantly less with Liraglutide *p* = 0.004	- Slower decline with liraglutide *p* = 0.01	- Significantly less with Liraglutide *p* = 0.003
**PIONEER-5** [65] Double-blind Randomized 26-week study and 5-week F/U	**Semaglutide** PO 3 mg, increase to 7 mg by week 4 and 14 mg by week 7	*n* = 324, DM II, CKD 3 Semaglutide 163 Placebo 161	- Week 26: Baseline 0.86:1.19	- Week 31:Baseline 1.02: 1.0, no significant	
**SUSTAIN 1–7** [55] Post Hoc Analysis	**Semaglutide** SQ 0.5 and 1.0 mg weekly versus compotators	*n* = 8416, DM II	- Significant decrease in UACR versus placebo 0.97 (semaglutide 0.5 mg) vs. 0.83 (1.0 semaglutide) versus 1.24 counterpart	- Decrease in eGFR with semaglutide until week 12, then plateau until week 30.	
**FLOW** [57] F/U Median 3.4 years	**Semaglutide** SQ 1.0 mg weekly Versus placebo	*n* = 3533. DM II, eGFR 50–75 mL/min/1.73 m^2^, Urine Albumin: Creatinine ratio (UACR) 300–5000; eGFR 25–50 mL/min/1.73 m^2^, Urine Albumin: Creatinine ratio (UACR) 100–5000 Semaglutide (*n* = 1767) Placebo (*n* = 1766)	- Decrease by 40% with semaglutide versus only 12% with placebo.	- eGFR less steep slope with semaglutide (*p* < 0.001) versus placebo	- Risk 24% less with semaglutide versus placebo (*p* = 0.0003) - Death reduced with semaglutide (*p* < 0.01) versus placebo
**REWIND** [62] Double-Blind, randomized F/U Median 5.4 years	**Dulaglutide** 1.5 mg SQ weekly versus placebo	*n* = 9901, DM II, Cardiovascular event or risk Dulaglutide (*n* = 4949) Placebo (*n* = 4952)	- Significant decrease in UACR with Dulaglutide versus placebo (*p* < 0.0001)	- No significant change in eGFR (*p* = 0.066)	- Significant decrease with Dulaglutide (*p* = 0.0004)
**AWARD 7** [63] Open-Label 52 Weeks	**Dulaglutide** SQ 1.5 and 0.75 mg versus Insulin Glargine	*n* = 577, DM II, CKD 3–4 Dulaglutide 1.5 (*n* = 193) Dulaglutide 0.75 (*n* = 190) Insulin Glargine (*n* = 194)	- No significant changes in UACR dulaglutide versus Insulin Glargine	- Higher eGFR with dulaglutide 1.5 mg (*p* = 005) and with dulaglutide 0.75 mg (*p* = 0.009) versus Insulin Glargine	
**AMPLITUDE-O** [64] Randomized placebo-controlled F/U Median 1.81 years	**Efpeglenatide SQ** 4 mg and 6 mg versus placebo	*n* = 4076, DM II, eGFR 25–59.9 mL/min/1.73 m^2^, + 1 cardiovascular risk Efpeglenatide 2717 Placebo 1359	- Decrease by 21%	- Increase by 0.9 mL/min/1.73 m^2^	- Decreased composite outcome with efpeglenatide 13% versus placebo 18.4% (*p* < 0.001)

### 5.4. Safety and Adverse Effects of GLP-1 (Table 4)

The safety of GLP-1RA drugs was the focus of several studies. SUSTAIN 10 [66] evaluated the safety of both semaglutide and liraglutide. Both drugs were administered subcutaneously. While semaglutide was shown to be superior to liraglutide in controlling diabetes, more GI side effects were noted with semaglutide.

**Table 4:** Adverse effects of GLP-1RAs.

Gastrointestinal; nausea, vomiting, diarrhea, constipation Neuropsychiatric events Acute kidney injury Sarcopenia Gall bladder disorders Retained gastric content aspiration Decreased bowel mobility and ileus Allergic reaction; itching, rash, tachycardia Dizziness Retinopathy Malignancy; Pancreas, Thyroid

Gerstein et al., who studied efpeglenatide in diabetic patients [64], listed several adverse effects using this drug versus a placebo. While most of the adverse effects noted were not significantly different than with the placebo, severe gastrointestinal events were reported in 3.3% in the treatment group versus only 1.8% among the patients receiving the placebo (*p* = 0.009).

The LEADER trial investigators [67] conducted a post hoc analysis of the LEADER trial, evaluating the safety of liraglutide. The authors focused on the effect of the presence (*n* = 2158) or absence (*n* = 7182) of CKD and the presence of macroalbuminuria (*n* = 966) or microalbuminuria (*n* = 2456) on the safety of the drug. The authors demonstrated that, while the serious adverse events were higher in the patients with CKD, no difference was noted in these adverse effects between the liraglutide-treated group and those receiving the placebo. No significant added adverse events were reported in the patients with macroalbuminuria versus those with microalbuminuria.

To evaluate the efficacy and safety of oral semaglutide, the PIONEER 5 trial [65] was conducted on patients with DM II and CKD 3 (*n* = 324). The trial was a multicenter randomized, double-blind study comparing semaglutide (*n* = 163) to the placebo (*n* = 161), with an outcome being a change in hemoglobin A1c, body weight, and safety. The trial team demonstrated that oral semaglutide was effective and superior to the placebo in lowering hemoglobin A1c and decreasing body weight and that, aside from GI symptoms, semaglutide was safe in this population.

Similarly, the FLOW trial showed no serious adverse events regarding using semaglutide compared to a placebo [56], and the SUSTAIN 1–7 trials demonstrated no added risk with semaglutide compared to the other drugs used in these trials [47,52,53,54].

In a recent Spanish study [68] focusing on the gastrointestinal adverse events in patients receiving GLP-1 RAs, a panel of researchers convened virtually to adopt practical guidelines to follow during the initiation and dose escalation of GLP-1RAs and to recommend measures to deal with the GI symptoms. The panel included three endocrinologists, two nephrologists, two primary care physicians, two cardiologists, two internists, and one diabetic nurse educator. They observed that nausea is the most common GI symptom. The other GI symptoms noted were vomiting, diarrhea, and constipation. The general and symptom-specific recommendations detailed by the panel included improving eating habits, exercising, and adequate hydration.

An alarming adverse event regarding GLP-1 was recently observed, namely non-arteritic anterior ischemic optic neuropathy (NAION). Hathaway et al. [69] conducted a retrospective matched cohort study of patients evaluated by neuro-ophtlamologists for 6 years. They noted that the diabetic patients receiving semaglutide (*n* = 194) had a higher cumulative incidence of NAION than those diabetic patients not on GLP-1RAs (*n* = 516) (*p* < 0.001). An accompanying invited commentary [70] recommended further studies to confirm the relationship between GLP-1RAs and NAION and to study the mechanistic pathways once a relationship is established.

## 6. Future Directions

The future directions for GLP-1RAs in DKD should focus on several key areas. Firstly, expanding the scope of the clinical trials to include a broader range of patient populations with varying degrees of kidney function, particularly those with more advanced stages of CKD, will be essential. Recent trials, such as the FLOW trial, have demonstrated the renoprotective effects of semaglutide, but further research is needed to confirm these benefits in broader populations and over more extended follow-up periods. Additionally, investigating the potential synergistic effects of combining GLP-1RAs with other renoprotective therapies, such as SGLT2 inhibitors, could improve clinical outcomes by targeting multiple pathways in DKD progression. Moreover, the preclinical studies suggest that GLP-1RAs exert protective effects beyond glycemic control, such as anti-inflammatory and anti-fibrotic actions, which warrants further exploration in non-diabetic CKD. Future research should also address the potential use of GLP-1RAs in earlier stages of DKD, focusing on prevention strategies rather than just treating established diseases.

The scope of the trials involving potential roles for GLP-1RAs could further include other disease entities for which preliminary studies have shown a potential role for GLP-1RAs. These diseases include patients with DM I, non-diabetic kidney patients, obese and/or hypertensive patients, and patients with metabolic liver diseases, peripheral vascular diseases, and neuro-degenerative disorders [71].

Lastly, as new GLP-1RAs and formulations (e.g., oral agents) become available, ongoing assessments of their long-term safety, efficacy, and impact on quality of life will be crucial to optimizing patient care.

## 7. Conclusions

Preclinical and clinical studies highlight the significant benefits of GLP-1 receptor agonists in improving renal outcomes for patients with diabetic kidney disease (DKD), mainly by reducing albuminuria. The data also confirm their safety in patients with diabetes and DKD. Multiple mechanisms likely contribute to their protective effects on kidney function. In summary, GLP-1RAs offer an exciting and valuable addition to our therapeutic options in the ongoing battle against DKD.

## Data Availability

No new data were created or analyzed in this study. Data sharing is not applicable to this article.
